# Adolescent pregnancies and girls' sexual and reproductive rights in the amazon basin of Ecuador: an analysis of providers' and policy makers' discourses

**DOI:** 10.1186/1472-698X-10-12

**Published:** 2010-06-07

**Authors:** Isabel Goicolea, Marianne Wulff, Miguel San Sebastian, Ann Öhman

**Affiliations:** 1 Department of Public Health and Clinical Medicine, Epidemiology and Global Health. Umeå University, Umeå, Sweden; 2 Department of Clinical Sciences, Obstetrics and Gynecology, Umeå University, Umeå, Sweden; 3 Umeå Centre for Gender Studies. Umeå University, Umeå, Sweden

## Abstract

**Background:**

Adolescent pregnancies are a common phenomenon that can have both positive and negative consequences. The rights framework allows us to explore adolescent pregnancies not just as isolated events, but in relation to girls' sexual and reproductive freedom and their entitlement to a system of health protection that includes both health services and the so called social determinants of health. The aim of this study was to explore policy makers' and service providers' discourses concerning adolescent pregnancies, and discuss the consequences that those discourses have for the exercise of girls' sexual and reproductive rights' in the province of Orellana, located in the amazon basin of Ecuador.

**Methods:**

We held six focus-group discussions and eleven in-depth interviews with 41 Orellana's service providers and policy makers. Interviews were transcribed and analyzed using discourse analysis, specifically looking for interpretative repertoires.

**Results:**

Four interpretative repertoires emerged from the interviews. The first repertoire identified was "sex is not for fun" and reflected a moralistic construction of girls' sexual and reproductive health that emphasized abstinence, and sent contradictory messages regarding contraceptive use. The second repertoire -"gendered sexuality and parenthood"-constructed women as sexually uninterested and responsible mothers, while men were constructed as sexually driven and unreliable. The third repertoire was "professionalizing adolescent pregnancies" and lead to patronizing attitudes towards adolescents and disregard of the importance of non-medical expertise. The final repertoire -"idealization of traditional family"-constructed family as the proper space for the raising of adolescents while at the same time acknowledging that sexual abuse and violence within families was common.

**Conclusions:**

Providers' and policy makers' repertoires determined the areas that the array of sexual and reproductive health services should include, leaving out the ones more prone to cause conflict and opposition, such as gender equality, abortion provision and welfare services for pregnant adolescents. Moralistic attitudes and sexism were present - even if divergences were also found-, limiting services' capability to promote girls' sexual and reproductive health and rights.

## Background

Getting pregnant during adolescence is a common experience for many girls, especially in low-income countries. Adolescent pregnancies have long been associated with adverse social and health outcomes for both the mother and children and pregnancy and childbirth related complications is the leading cause of death for girls aged 15-19 [1-4]. However, many qualitative studies also evidence that adolescent pregnancy can be perceived as a positive experience [5-13].

In Ecuador, 20% of girls get pregnant during adolescence, with marked disparities between adolescent girls of different educational levels, socio-economic status, and geographical regions: while 43% of illiterate adolescents experience pregnancy, only 11% with secondary education do. Regarding geographical areas, the highest adolescent fertility rates are found in the amazon basin [14]. As a response to this situation, the country has been implementing a National Plan for Adolescent Pregnancy Prevention since 2007. The Plan draws on already existing national policies and laws relating to adolescents and sexual and reproductive health and rights, such as the National Policy of Sexual and Reproductive Health and Rights, the Free Maternity Law, the Law Against Violence Against Women and Family, the Law for Sex and Love Education, the Child and Adolescent's Code, and the Youth Law. The Plan focuses on the implementation of "adolescent-friendly services" through the already existing public health facilities, alongside the strengthening of sex education in schools and the promotion of adolescents' participation [15].

### The rights approach to adolescent pregnancies

The connection between adolescent pregnancies and sexual and reproductive rights seems evident: access to contraceptives is a cornerstone in the prevention of unwanted pregnancies, while access to abortion services is crucial for managing them. Access to maternal and neonatal health services can minimize many of the risks associated with adolescent pregnancies, and protected sexual intercourse is the way for preventing both unwanted pregnancies and sexually transmitted infections, including HIV. Gender inequality can work overtly through coerced sexual intercourse resulting in an unwanted pregnancy or, more subtly, through reinforcing gender constructions that overvalue marriage, pregnancy, and motherhood as the most feasible life projects for girls.

Looking at adolescent pregnancies from a rights approach aims not merely to prevent adolescent pregnancies, but to understand why they occur, both unwanted and wanted. The rights approach stresses the connection between individuals' freedom and their entitlement to a system of health protection. Freedom is conceptualized not merely as the right to act, but as the capability to act, the actual power to make choices [16-19]. Gender inequality strongly limits women's and girl's capability to make free and informed decisions regarding sexuality and reproduction. Entitlement to a system of health protection is conceptualized not only as the state's responsibility to ensure an adequate array of health services, but also its responsibility to take measures to reduce the inequities that negatively affect the health of certain groups and individuals - the so called "social determinants of health" [19-23].

A rights approach to adolescent pregnancies aims to look for ways to strengthen girls' freedom over their bodies and lives, and to entitle girls to an adequate network of reproductive and sexual health services and a healthy environment that ensures that girls have sex, get pregnant and become mothers only when they freely and safely decide to [24-30]. Policy makers and service providers play a crucial role in the development of such a network, and also-although to a lesser extent- on promoting a healthy environment concerning the social determinants of girls' sexual and reproductive health [25,28,29,31]. Policy makers' and service providers' discourses on adolescent pregnancies and sexuality are not just a neutral reflection of the situation but in fact construct the plans, actions and services that will be available for supporting adolescents' right to enjoy safe and pleasant sexuality and reproduction [32]. Exploring those discourses could shed some light on the way adolescents' sexual and reproductive health programs are constructed and delivered, and, consequently, could contribute to strengthening them.

The present study is part of a broader research exploring adolescent pregnancies in the amazon basin of Ecuador (Orellana province). Previous studies have stated that gender structures constrain girls' agency, reducing their capability for making decisions regarding their sexuality and reproduction. The use of contraceptives is a protective factor, but misinformation and secrecy regarding those issues prevent many girls from adequately using contraceptive methods [33]. The aim of this study is to explore policy makers' and service providers' discourses concerning adolescent pregnancy, and to discuss the consequences that those discourses have for the exercise of girl's sexual and reproductive rights' in the province of Orellana.

## Methods

### Study area and participants

The study was conducted in the province of Orellana, in the amazon basin of Ecuador. With 103,032 inhabitants and 22,500 km2 of rainforest, it is a mainly rural population (70%), with a significant indigenous subpopulation (30%). It is a young population, comprising 27% adolescents.

In Orellana, the percentage of pregnant adolescents is almost 40%. Forty-five percent of adolescent pregnancies are labeled as wanted, which is a high rate when compared to wanted pregnancies among other age groups in Orellana (40% for women age 20-29, and 32% for women age 30-39), but extremely low when compared to countrywide rates of wanted adolescent pregnancies (70%) [14,34]. Adolescent pregnancy in the province has sometimes been linked to sexual abuse during childhood-adolescence, poverty, parental absence, early sexual debut and lack of use of contraceptives during first sexual intercourse [33].

For this study, we used eleven individual interviews and six focus group discussions to explore discourses concerning adolescent pregnancies of policy makers and service providers of the health, educational and welfare sectors at the district and provincial level in the province of Orellana. We mixed focus group discussions and individual interviews in order to explore both group dynamics and interaction between providers (focus groups), and individual experiences and perceptions (individual interviews). Policy makers included such individuals as the head of the Sex Education Program, the head of the Adolescents' Health Program, and the head of the Youth Program of the Ministry of Social Welfare. Service providers included teachers working with sex education, social workers, and health providers (medical doctors, nurses and midwives) working on reproductive health issues and/or adolescents' health. Some of the providers had work experience of intimate partner violence and sexual violence. We first conducted focus group discussions with service providers, and afterwards we interviewed individual policy makers, with whom focus group discussions were more difficult to arrange. Regarding gender, seven women and four men participated in the individual interviews, while fourteen women and sixteen men participated in the focus group discussions.

In Orellana there is no local ethics committee but several actions were taken to ensure compliance with ethical research principles, including the ones stated in the Helsinki Declaration. Approval of the study design was obtained from provincial authorities, and informed consent was sought from all the participants in the interviews and focus group discussions. Participants were advised that results would be reported as an aggregate with exemplar quotes, and that no individuals would be named. Measures to ensure confidentiality included erasing names and workplaces on transcripts. Anonymity was not achievable in the focus group discussions, but the moderator stressed the importance of not disclosing the content of the discussions outside the focus groups. Two workshops for discussing preliminary results with providers and policy makers in Orellana have already been held, with the aim of starting the process of disseminating the results in the community.

### Data collection

The first author (IG) conducted all the individual interviews and moderated the focus group discussions. IG has been working in the area for ten years, and that facilitated access to participants. Two of the other authors were not familiar with the setting, and that helped to keep the balance between internal and external perspectives. The discussions lasted from 47 to 94 minutes, and the number of participants ranged from three to seven, with a total of 30 participants. The individual interviews lasted from 25 to 65 minutes. Participants were approached by the first author or by health and education provincial authorities; none of the approached persons refused to participate. Both the individual interviews and the focus group discussions were conducted in different locations depending on the participants' wishes. We took into account hierarchical differences in terms of profession and position, in order to arrange groups where participants would feel free to give honest and full opinions. Group participants were from both sexes; sometimes groups were mixed-gender, sometimes not. When groups were mixed, it did not seem to discourage any interaction within the group. Since the questions focused on their opinions and experiences as professionals and not on their personal lives, the sensitivity of the topic was reduced and both women and men participated just as much in the discussions. The majority of participants knew each other. Since Orellana is a small province with not many people working on issues related to adolescent pregnancies, anonymity was impossible to attain. For policy makers, individual interviews were held to avoid power imbalance within the group, and because it was more feasible to reach them in that way. The process ended when saturation was reached (no additional information emerged from the interviews).

At the beginning of the individual interviews and focus group discussions, the interviewer explained the aims of the study and asked permission to record the session. No one but the interviewer and the participants was present. Participants were also asked whether they could be contacted again for clarification if needed, and all agreed.

Both the individual interview and focus group discussion guides followed an open format, meaning that the guide contained a few themes that were explored, but no specific queries. After some common introductory questions, the interviewer asked about participants' perceptions of adolescent pregnancies in Orellana. For this study, we used the World Health Organization's definition of adolescent pregnancy as "any pregnancy from a girl who is age 10 to 19" [35]. Participants were encouraged to talk freely and, when in a focus group, to interact with each other and not just answer the interviewers' questions. Issues that were covered related to participants' views and experiences concerning adolescent pregnancies, such as judgments about the nature of individual pregnancies as to whether they were problematic, personal and professional experiences with adolescent pregnancies, and perceived causes and consequences of adolescent pregnancies. Other issues that were covered included participants' opinions concerning the partners of pregnant adolescent girls, their families, and whether there was any relationship between adolescent pregnancy and other reproductive and sexual health issues such as violence, abortion, contraceptives, sex education, health services, social services. The interviews ended with questions concerning the activities that institutions were implementing on adolescent pregnancies prevention, and participants' opinions on what should be done. We followed-up relevant issues that emerged in subsequent interviews following an emergent design [36].

The interviews were performed in Spanish, which was the mother tongue of the interviewer and of all of the respondents. They were tape recorded and fully transcribed, including silences, laughter, or other signs of emotion. To ensure confidentiality all names were erased. Transcriptions were entered into Open Code 3.4 for managing the analyzing process [37].

### Data analysis

The authors read the interview transcriptions several times and coded them using the original Spanish text. Afterwards, interviews and codes were read again, looking for statements regarding adolescent pregnancies and subjects who personified them-such as the stereotype of the impoverished and single adolescent mother-, the means through which statements acquired their authority - such as references to experts' statements and national statistics - and the policies and practices that emerged from the discourses. The emergence of defiant discourses was explored as well. We looked for issues that were overtly expressed or even challenged, but also remained alert to issues that remained unchallenged or even unaddressed until directly raised by the interviewer. Though there may have been differences and similarities between different types of respondent-men and women, for example- we focused only on the overall discourse on adolescent pregnancy without such a breakdown.

Finally, all the above information was analyzed looking for "interpretative repertoires". Interpretative repertoires refer to the clusters of meanings that people utilize to built up their arguments, in a way that seems sensible for them; in Wetherell and Potter's words they are "the building blocks used for manufacturing versions of actions, self and social structures in talk" (Wheterell and Potter, p. 90) [38]. This perspective to discourse analysis follows Foucault's approach since it explores how people make sense and how they justify the construction of the repertoires, but it also follows critical discourse analysis in the sense that it looks into the possible consequences of the repertoires on peoples' lives [39].

Interpretative repertoires acknowledge that variability, inconsistencies and contradictions occur within everyday discourses. During the same interview, the interviewee might make one statement at one point, and a completely contradictory one later on [38-40]. However, when looking for interpretative repertoires we do not explore each interview as an isolated item, instead we look across all the interviews - with theirs contradictory statements - to find recurring consistent units; those recurring consistent units constitute the so called "interpretative repertoires" [40]. In our case, we looked for the interpretative repertoires that addressed adolescent pregnancies in Orellana, and the effect of those repertoires on the exercise of sexual and reproductive rights by adolescent girls living there, intermediated by the policies and services that those repertoires produced for adolescent pregnancies prevention and management.

## Results

The four interpretative repertoires that emerged from the analysis were: sex is not for fun, gendered sexuality and parenthood, professionalizing adolescent pregnancies, and idealization of traditional family.

### Sex is not for fun

The participants stated that issues related to sexuality should be approached with caution. Sexual intercourse could be dangerous because of the risk of getting a sexually transmitted infection and the risk of an unwanted pregnancy. But especially, sexual intercourse could be dangerous because it could be "misused" or "overused". Dangerous sexual intercourse was described as having too many sexual partners, having sex outside a formal union, having sex without a romantic relationship or having sex too young, and thus not being fully aware of all the implications of having sexual intercourse. The words used -and the avoidance of explicit terms- for referring to sexual intercourse among girls, created the impression of doing something illicit:

*"Then all this pushes adolescents to become... *[sexually active] *what they should not become at that age..." *(Hospital social worker, woman, FG social-health workers).

Adolescents were portrayed as unable to take responsibility and unable to understand all the consequences of sexual intercourse, while adults were perceived as more capable of doing that, and mature enough for engaging in sex. That perception conflicted with the acknowledged fact that many pregnancies of adolescent girls were fathered by adult "mature" men.

*"Yes, I mean it is not a fixed rule that he is always older or adult, but...., yes, it's a tendency... a big tendency that one can observe...isn't it?... Ten years of difference... five years... *[...] *I mean, in the majority of cases there is that tendency that women are more attracted towards older men..., and then, because of this... men..., it is maybe easier to get involved with a younger girl..." *(Hospital psychologist, man, FG social-health workers)

Participants agreed on the need to implement sex education for adolescents. The approaches to sex education varied, but the stress was mainly put on sex education as a way of preventing the adverse consequences associated with sexuality and sexual intercourse, and very little on acknowledging the pleasant aspects of sexuality. The focus was put on the sexual act, and not on its characteristics: if it was protected or not, if it was consensual or not, if it was enjoyed or not. As the focus was placed on adolescent pregnancy as a consequence of premature sexual intercourse, more than as a consequence of unprotected sex, the approach to contraceptive use among adolescents was inconsistent. Contraceptive use was thus not perceived as the key to adolescent pregnancy reduction. Focus was on delaying sex more than on using contraception. Contraceptives were perceived by some participants as a "double-edged weapon" that could lead to promiscuity or increasing sexual intercourse among adolescents.

*"With contraceptives there is a double risk... *[...] *are we going to prevent all the consequences of early initiation of sexual relations? *[...] *No, instead we are going to enlarge and increase the problem at the national level. *[...] *For me contraceptives are like trying to cover up the sun with a finger*" (Hospital gynecologist, man, FG health professionals)

As a consequence, messages regarding the promotion of contraceptive use among adolescents were ambivalent: information should be given, but not full access to the means; adolescents should be free to use contraceptives, however it would be better if they delay sexual intercourse. Messages also stressed that adolescents should use contraceptives if they were not capable of "controlling" their sexual desires, although they should control themselves and engage in other kinds of relationships. It was seen as acceptable for adolescents to use contraceptives, but many times they were perceived as unable to use them correctly and prone to overuse of some methods like emergency contraception. Finally, sentiments urged a reduction in the number of adolescent pregnancies, but society was perceived as being unwilling to accept full access to contraceptives for adolescents.

Pregnancy was perceived as a reminder of past sexual intercourse, it was the "body of the crime", as one participant expressed it. For instance, a pregnant student was a visible sign of sexual activity, in an environment where sexual intercourse should not be taking place. Participants expressed how schools' approach had changed compared to a couple of years ago when many pregnant students were discharged for fear of an adolescent pregnancy epidemic at the school or damaging the school's reputation - though this was seldom the case, and attitudes had become more supportive. In this transitional period from banning to supporting, some very strong attitudes backing pregnant girls' right to education coexisted with perceptions of pregnant girls as something that should be "hidden". At the same time that schools maintained discriminatory acts, individual teachers might choose to fight for the rights of the pregnant students.

*"What happens at my school,...and it is a public school, is that they don't allow a pregnant girl to finish the academic year. I mean, because it has two schedules *[day and night], *the pregnant girl is sent to the night schedule. That's how the school authorities camouflage it" *(Teacher, man, FG teachers)

*"But, colleague, you should have asked that question to the authorities at your school: 'And what would happen if that pregnant girl had been your daughter?'... Just to see how will he look at it from that perspective... We do those things *[expel a pregnant student], *because they do not personally affect us... *[...] *Then, that's the task that we who are working in the educational field have: we have to invite our colleagues to reconsider their positions... we have to be aware that those adolescents, those youngsters that are in process... they are part of us... and we have to stand for their rights" *(Teacher, woman, FG teachers)

A way of sanctioning sexual intercourse was ensuring that it was performed within a committed relationship, and the institutionalization of this commitment was a formal union or marriage. Thus, sexual intercourse between adolescents -or between an adolescent and an adult-somehow acquired legitimacy if it was done within a formal union.

*"She may be a child, but if she has a husband with her, then nothing happens. People might say, 'what a pity she is so young; but, she has a husband'. And she could be older, but has a baby anyway without a husband; she too has problems. I mean, it is problematic to get pregnant without a husband..." *(Teacher, FG teachers)

### Gendered sexuality and parenthood

Gender influenced the way sexuality and sexual intercourse were perceived. While men were expected to be sexually driven, women were expected to be less in need of sex. Sexual relationships were modulated by these assumptions: men would always try to get sex with girls, and girls should be hard to conquer. Whenever a man had sex it must have been because he wanted to, whenever a girl had sex it could have been because she was talked into it. Boys had sex because that was their nature, girls had sex as a way of expressing compromise.

*"Ah..., let's say that when a man is 30 years old, he..., then, what happens?... that men...the...what..., it is in man's nature to have sex fixed inside his head and nothing more. So, the first thing he is going to do is look for a woman... *[...] *he doesn't measure the consequences" *(hospital gynecologist, man, FG health professionals)

*"Sometimes girls let themselves be persuaded, seduced, ...that crazy youth who deceives them...This is what happens to those who don't attend school. And after being seduced, they get pregnant" *(Head provincial sex education program, man).

Responsibility for preventing sexual intercourse, preventing pregnancy, preventing sexually transmitted infections was placed on the shoulders of girls, since men and boys were perceived as unreliable, not caring at all, and even intentionally wicked.

"[Regarding why boys don't use contraceptives] *Sometimes, it is because of lack of information... even men! *[...] *But many times I think is just wickedness, just to say 'I slept with that girl, I don't care if I get her pregnant! It's her problem"' *(Medical doctor working with gender-based violence, woman)

Only providers working with gender-based violence raised the issue of sexual violence. According to them, sexual intercourse for girls often took place within an environment of coercion or overt violence. Males were perceived as violent and abusive, taking advantage of girls' need of love and affection not worried about the possibility of getting them pregnant or infected. The "sugar daddy" phenomenon-dating and sexual intercourse between adult better-off men and local adolescent girls in exchange of gifts or payment for education- was perceived as frequent and labeled as sexual abuse by them. On the other hand, providers not familiar with gender-based violence in their practices, were less stringent in their definition of sexual abuse, and considered sexual intercourse between a girl and an adult not as a sexual offense but merely as a prank.

*"The girl used to go to..., in that house some workers from the oil company rented rooms, and the girl, she was 12, or 13, she was only in her last year of primary school. And the girl well, since, since she didn't have money for buying clothes she went to the men's rooms and let them touch her... *[...] *Her grandparents even encouraged her. And one of these guys decided to risk having sex with this girl... *[laughs]. *But the money was not for the girl, but for the grandparents*" (Teacher, man, FG teachers)

Sexual intercourse was constructed within gender norms, and the same applied to parenthood. Gender norms influenced parenthood and sexuality in opposite directions: while men were "naturally" prepared for sex, they were unreliable as fathers, and while women were not sexually driven, they made sacrificed and selfless mothers. Men and women, sexuality and parenthood were opposed elements:

*"Men have the idea that the only one responsible for pregnancy is the woman, OK? It is because ****she****didn't use contraception" *(Medical doctor working with gender-based violence, woman)

For girls, adolescent pregnancy was perceived as dangerous because of the potential obstetric and neonatal complications as well as the socioeconomic impact. However, providers agreed that once pregnancy occurred, the girl should be encouraged to endure it, leaving limited room for abortion.

*"In this case I ask her, 'Do you want to see your baby?' ... Since she tells me that because she has not a belly, there is nothing there... I told her: 'Let's have a look...' You don't feel it because he is still little, and there is all this liquid surrounding him...' Imagine after seeing him like that..., 'Well I respect your decision, if you want to have an abortion is your decision, OK?... If you are going to have one, do it in a safe place, with all the warrants, to ensure that you do not put yourself in danger... *[...] *'Then she stared..., with tears in her eyes... *[...] *and I asked her: 'Do you know the father of the baby?' She told me that he has been a classmate, that they went to a party, he made her drunk and sexually abused her... *[...] *And she told me: 'Doctor, being like this I did not want to have an abortion' *[...] *From that point on she was a patient that did not miss a prenatal control... Now she takes the baby to the controls... she loves her baby!" *(Medical doctor working with gender-based violence, woman)

Fathers were absent in the discourse of adolescent pregnancies in Orellana. Efforts to engage fathers in the care and economic support for the babies were lacking within adolescent pregnancy programs. From this repertoire, the subject of the adolescent mother was constructed as single, lonely, victimized, and economically deprivated since she had not a partner supporting her. At the same time the adolescent mother was expected to sacrifice all for her baby, because she couldn't count on the father, and even the society did not dare to make the father accountable.

"[When asking regarding the fathers] *They leave..., they disappear... It's men's irresponsibility as well*" (Hospital gynecologist, man, FG health professionals)

*"They are the mothers..., they are the ones who have to wake up at night, isn't it so? They have attained a greater responsibility than what they had before..., yet they... continue... being... adolescents. Obviously no..., as I said in the beginning..., they aren't going to be able to go partying all weekend or traveling all week, because they have to take care of their child*" (Hospital psychologist, man, FG social-health workers)

### Professionalizing adolescent pregnancy

Knowledge regarding adolescent pregnancies in Orellana was compartmentalized and quite hierarchical. For many years adolescent pregnancies had been a common experience within this province, dealt with by families and communities in different ways but now they became labeled as a health and social problem, the field of professionals.

*"Only recently during the last few years, people are talking about adolescent pregnancy... we have known about it for many years but nobody said anything, nobody said anything, what should we do? ...Let's say that if there is going to be a campaign to prevent adolescent pregnancy, let's say that people should know, should talk about it..." *(Provincial ministry of welfare officer, man)

The construction of adolescent pregnancies as a problem came from the national level, and has produced a number of documents, plans and policies, that also contributed to reinforce the conceptualization of adolescent pregnancy as a problem that should be approached by professionals. Providers and policy makers used a technical language filled with scientific terms-rates, percentages, risks, obstetric complications, adolescent-friendly services, subsequent pregnancies- and references to scientific literature, "experts" opinions, situation analysis, national plans and policies, and professional experience.

Participants distanced themselves from the ones who were "suffering" the problem-lay people, uneducated people, people from the bush, poor people. Since professional knowledge is acquired through years of formal education, it also lead to segregation between the adolescents -who were "suffering" the problem of adolescent pregnancies- and the adults -who were technically qualified for "dealing" with the problem. This, adult-centric approach had consequences in the way providers approached adolescents. They were not treated as autonomous individuals but as incomplete and immature, unable to make their own decisions.

*"It is precisely with that kind of patient *[pregnant girl] *that it is better to do the consultation together with her mother or father; maybe the mother will have more influence on the girl... I mean, to tell her 'this is how you should behave, this is how you shouldn't behave"' *(Hospital midwife, woman, FG health professionals)

It has to be acknowledged that providers all seemed interested and willing to help adolescent girls. Many were already seeing pregnant adolescents during consultation or at school, and were worried for them, especially when they didn't follow the advices that were intended to help them. In that sense, adolescents were classified into "good patients" -the ones who comply with the doctor's recommendations- and "bad patients". Many times providers talked about "their patients" or "their adolescents", in an attempt to express their closeness with them. However, this could also be seen as a possessive approach to adolescents. They used diminutives as "mamita" (mommy) or "mijita" (my little girl), which could be regarded as a way of gaining trust, but also as a way of patronizing girls. Confidentiality and privacy were felt as necessary to ensure adolescents' access to services. However, participants recalled many occasions when those were completely disregarded:

*"I first approached the girl *[a student at his institution], *'hey my little girl' I say 'isn't it possible that you are pregnant? ... I swear that I won't mistreat you'...What can I say? 'No, my little girl, seeing, seeing your changes...., you are pregnant my love.' 'No' she told me, 'they keep on saying that I am pregnant, but I am not.' Well, nothing happens. I go and buy the pregnancy test. I tell her, 'My little girl, let's go get you a pregnancy test.' And just at that moment the concierge was there so I asked her to help me with a urine test [of the girl], and I did the test... I took it and two lines appeared... There I tell her, 'See, my love, you are pregnant"' *(Teacher, man, FG teachers)

Youth participation was strongly supported by the providers, however, the way it was understood varied. For many, adolescents' participation meant inviting adolescents to participate in plans and programs developed by professionals, since adolescents' opinions on the issue were not perceived as important as professionals' judgments. Others, mainly the youngest participants, talked of the need, and lack, of a more active and critical adolescent participation at all levels of planning, implementing and monitoring.

*"Regional workshops have also been held... where mainly... where... where young people have said how they want health providers to treat them... *[Representatives from] *all the institutions attended and young people said that they want to be treated like this and like that... *[...] *And health care providers... said 'that's too idealistic, that can't be done'... So, it was as if...., as if they said ... 'that's impossible *[...], *of course, we are here to listen to your proposals but..." *(Youth representative of the Andean Committee for Adolescence Pregnancy Prevention, woman)

The adult professionals did not constitute a homogeneous group; there were hierarchies between the different professions: the medical profession was perceived as responsible for adolescent pregnancy prevention and management, and medical knowledge was perceived as the most important for dealing with adolescent pregnancies. The expertise of educational providers, social providers and, especially, providers working with gender-based violence, was perceived as complementary to the medical discourse when dealing with adolescent pregnancies and adolescents' health. Doctors were perceived as the most competent in dealing with sexuality and adolescents' health issues, even if many adolescents' health complaints were not defined as medical problems. Thus the possibility of pregnancy due to rape, or the connections between adolescent pregnancies and violence were only expressed by providers working with gender-based violence. Sexual abuse outside, but especially inside, families, was perceived as very frequent, but the connection with adolescent pregnancies was seldom expressed. Participants working on gender-based violence, felt left out of the planning and implementation of programs addressing adolescents' reproductive and sexual health and pregnancy prevention.

*"It could be that yes, that *[health care providers] *are not trained or that they don't have the approach of *[gender-based] *violence, sexual violence... and they just focus on the pathological aspects, treat the disease and that's all. So, look here, these people need to be trained and just refer the patient nothing more..., that would be great *[...] *But, unfortunately every time that we want to raise the issue even slightly... they look at us ... I don't know what to think anymore... I, truthfully, I have chosen to not say anything when I go to the meetings" *(Head of NGO working with gender based violence, woman).

### Idealization of traditional family

Another important repertoire was the idealization of traditional family. Family was perceived as key to adolescent pregnancy prevention, and fundamental for adequately raising children and adolescents, by socially reproducing the values that were perceived as good. Even if the medical sector was perceived as more capable of dealing with adolescent pregnancy, the family remained the most responsible for what might happen to adolescents, in terms of sexuality and reproduction.

*"Because education is based there *[in the family]. *We cannot expect that adolescents will be taught about values and principles and reproductive health at school or high school or the university because these are values that they learn at home. If adolescents are not taught these values at home, it doesn't matter how much they give them at school, they will never learn them" *(Hospital gynecologist, man, FG health providers)

The "traditional" family was perceived as a shelter, a positive environment for learning good behavior. The "traditional" family was described more as a heterosexual nuclear family, than as an extended family, following the patriarchal model of father, mother and children, all caring, but also all knowing each others' hierarchical roles: parents deciding, children obeying. Single-parented families were perceived as defective and at higher risk of raising problematic children or girls who got pregnant early.

*"For example, father, mother and children... well structured. Father goes to work... that would be a modern family...But, the father migrates to Spain, the mother is left alone... there is no order. The father is father and he is always going to be setting an example for his sons and daughters... and the same applies to the mother" *(Hospital midwife, woman, FG health professionals)

Traditional family was perceived as fundamental to the survival of the Ecuadorian way of life, and thus "different" ways of constructing families, or challenges to those traditional values (related to family formation, sexuality and reproduction) were presented as an undesirable foreign influence that should not be followed by Ecuador. There were many opinions in favor of strengthening and educating families as the key to adolescents' proper development, and centering responsibility for sex education in the family.

*"Society has put a lot of emphasis on the school, and this is not the solution... *[...] *We should join together to rescue the families... *[...] *I think that would be the most effective and suitable... Because the girls who get pregnant, they are girls who are cold, empty, who have not experienced love, affection...and who better than a father, than a mother to give them this affection?" *(Responsible for students welfare at a secondary school, woman)

At the same time, participants acknowledged that families, even when fulfilling the characteristics of the "traditional nuclear model", were sometimes dangerous for adolescents' health and proper upbringing. The interviews were full of perceptions and experiences of violence and sexual abuse within the families. There were comments regarding families that forced adolescents to engage with older men for money; domestic violence against children and adolescents was perceived as a strong reason for girls running away from home, or seeking a partner as a way of escaping from abusive families; incest was perceived as common.

*"Yes..., they knew..., they knew from the first day. From when the girl tells me that she has been sexually abused until she finds out that she is pregnant, it is a long time and her mother knew! 'And even though you knew' I told her 'even though you knew, you were not able to bring your daughter so that they could help her' *[...] *It was a girl who suffered from sexual abuse for two years..., the husband, the father has been sexually abusing her..." *(Medical doctor working with gender-based violence, woman)

## Discussion

Within the rights approach, the capability of girls to exercise their sexual and reproductive freedom is strongly linked to their entitlement to a system of sexual and reproductive health protection that ensures both an array of relevant services and a healthy environment [21,41-43]. Providers and policy makers' repertoires do not neutrally describe what goes on regarding girls' sexual and reproductive health, but in fact have implications and consequences for the way they approach those girls and the actions they take to deal with adolescent pregnancies and adolescents' sexual and reproductive health [32,39,40]. In this study those repertoires were dictating the array of sexual and reproductive health services that would be available for girls in Orellana and the way they were delivered. To some extent they also influenced girls' sexual and reproductive freedom, and the social determinants of sexual and reproductive health beyond the provision of health care.

The "sex is not for fun" repertoire could be perceived as a starting point for stressing the urgency for implementing sexual and reproductive health services for adolescents. Since adolescents' reproductive and sexual health and rights have not been high in the public policy agenda of many Latin American countries [25,30], stressing the harmful, and preventable, consequences of sexual risk-taking for adolescents and the society as a whole, could be an argument for addressing the lack of interest in this issue. Providers' acknowledgement that girls should be entitled to a network of reproductive and sexual health services (that included access to sexual and reproductive education and information) could create an opportunity for strengthening sex education programs in Orellana. However, the "sex is not for fun" repertoire might also shape those services in a way that constrains girls access to "relevant" information: contradictory messages regarding contraceptive use might discourage girls from using them; providers' difficulties with managing selected sexuality issues might lead to incomplete or misleading sex education programs; services that do not take into account issues of gender inequality or gender-based violence and sexual violence might fail to fulfill the reproductive and sexual health needs of girls. This way of providing biased information does not seem to be the best, since several studies show that the greater impact on adolescents' sexual behavior comes from comprehensive information on sexuality and not from abstinence-only education [44-47].

The network of reproductive and sexual health services that the gendered sexuality and motherhood repertoire constructed was one oriented towards girls and women, thus reinforcing the idea that reproductive matters were exclusively women's/girls' responsibility. Moreover, since girls were not expected to be interested in sex, but in reproduction, issues regarding sexuality might not be addressed as importantly as issues regarding contraceptive use and maternal and child health. As a consequence, girls might end up being entitled to health services that reinforce their responsibility regarding pregnancy - including pregnancy prevention- and motherhood, and deny girls' right to be sexually active and well-informed and protected. This "mother-nization" of reproductive services for girls has also been described by Varea in a study in Quito's Isidro Ayora Maternity [48].

Providers acknowledged that illegal and unsafe abortions were common among adolescents, and that pregnancy due to sexual abuse was common. However, abortion had no place within the array of services girls' should be entitled to in Orellana. Pregnant adolescents were persuaded to accept, love the fetus and make sacrifices for its wellbeing, even when pregnancy was the product of unwanted sexual intercourse or rape.

The array of services that girls were entitled to also left out the provision of support for child-raising, such as childcare services, maternity leave, and measures to ensure paternal compliance with their economic responsibilities; those were not available within the array of services under the state's responsibility, but remained the individual's - mainly the woman's-responsibility.

If we conceptualize freedom not just as individual liberty, but as the actual capability to make choices, then it does not only depend on the existence of a network of policies and laws, but also on the degree of implementation and the impact those policies have on individuals' capability to exercise their rights. Both the "sex is not for fun" repertoire and the "gendered sexuality and parenthood" repertoire curtailed girls' freedom to exercise safe sex by displaying a normative code that disapproved of sexual intercourse for girls before they established a formal relationship, did not straightforwardly encourage girls to use contraceptives, and reinforced submissive, dependent, and obedient attitudes. This normative code is produced and reproduced by the machismo-marianismo system that emphasizes men's dominance while encourages women's submission, chastity, and self-sacrifice [32,49-51]. This normative system, alongside Ecuador's Penal Code - in Ecuador abortion is legally permitted only when the life and health of the mother are at risk and when pregnancy is due to rape of a mentally disabled woman- curtailed girls' freedom to decide upon the continuation or termination of their pregnancies, by stressing motherhood as women's natural responsibility.

The "professionalizing adolescent pregnancies" repertoire also greatly influenced the way sexual and reproductive health services were provided. Even if providers were truly well-intentioned, the way they approached adolescent girls -and boys- could be constructed as paternalistic and patronizing: girls were not really heard but advised, confidentiality could not be ensured and professionals assumed they had the right to decide what was best for the patient. Those attitudes of health providers have been found to be ineffective for reproductive and sexual health promotion, and contribute to further disempowering girls. Instead, the encounter between the provider and the girl could be reconstructed differently, as an opportunity for helping girls gain agency and freedom towards their sexual and reproductive life [29,31,52-54].

Since adolescence was viewed by professionals as an underdeveloped stage, youth participation in decision making in this area was largely non-existent. However, dissident voices were also present: some of the youngest policy makers who participated in our study had a critical view regarding youth participation, asking for youth involvement at all levels of the decision making process. This could be an encouraging sign in Orellana, since evidence from other settings has established that youth participation in developing policies and programs addressing their needs is effective in implementing more meaningful interventions, and it is also a basic principle of the rights' approach to sexual and reproductive health [24,25,27]. The hierarchical division of expertise left out, (or at least almost left out), issues and providers working in gender-based violence. As many studies show, sexual abuse and intimate-partner violence are vital problems limiting girls' freedom to enjoy sexual and reproductive rights and any system of health protection should take them into account as central features [24-26,28,33,48].

The idealization of institutions such as marriage and family might also constrain girls' freedom to exercise their sexual and reproductive rights, by overseeing girls' vulnerability to sexual abuse, violence and other violations of rights of girls living within those "safe" institutions [55-57]. "Familism", a cultural value typical in Latino societies that "weights on the interdependence among nuclear and extended family members for support, emotional connectedness, familial honor, loyalty, and solidarity" (Munoz-Laboy, p. 773) [58], could have positive consequences in the way of acknowledging the important role of families influence on girls' sexuality and sexual behavior. It could have a positive impact on promoting healthy behavior, but it might also foster very restrictive norms for controlling girls' sexual freedom, especially when embedded within the machismo-marianismo system [32,58].

Providers' and policy makers' repertoires determined the areas that the array of sexual and reproductive health services should include, leaving out the ones more prone to cause conflict and opposition, such as gender equality, abortion provision and welfare services for pregnant adolescents. In that way, sexual and reproductive services for adolescents could have more chance of being implemented, but it raised questions regarding how adequately such services would address adolescents' sexual and reproductive needs. Moreover, even in the areas that were part of this narrowed array of services, patronizing and hierarchical attitudes might further diminish the positive impact of the provided services.

In this study, issues related to gender relations were preeminent. Women's subordination is a crucial social determinant of poor sexual and reproductive health [19,20,59,60]. Institutional gender regimes interact with the wider gender order, and could reinforce gender based inequities or contribute to challenge and resistance [61]. The present study evidenced that, in Orellana, gender regimes of reproductive and sexual health services mainly reinforced girls' subordination, although attempts to resist and challenge were also present. On the one hand, services were provided in a hierarchical way that reinforced young girls' disempowered position and devalued their knowledge and experiences. Policies that did not take into account young single women's needs for economic support and child-care further constrained their possibilities of being independent. On the other hand, the fact that some providers challenged the discriminatory gender regimes of their own institutions, acknowledged the problem of sexual and intimate partner violence within marriages and families, and struggled to ensure adolescents' participation, established opportunities for making this rigid gender and generational order more prone to change.

This article opens the discussion regarding how policies and services might best contribute towards the realization of adolescents' sexual and reproductive rights. Girls' reproductive and sexual freedom could be enhanced by the provision of adequate, unbiased information and services -especially regarding contraceptives- that comply with the standards of availability, accessibility, acceptability, good quality, relevance and equity. The repertoires emerging from this article also point to the relevance of critically questioning the consequences of moralistic attitudes that stigmatize girls' sexual activity and focus on the prevention of girls having sexual intercourse, instead of the prevention of unprotected, coerced, uninformed, and unpleasant sexual activity. It would be interesting to explore mechanisms to reconstruct masculinity and parenthood in a more constructive way, and to foster men's/boys' responsibility towards their offspring. Services might play an important role in achieving that if policy makers conceive boys and men, not only as a menace but as an opportunity for improving girls' -and their own- reproductive and sexual health. At the same time, it appears important to reconstruct motherhood not as a woman's destiny, but as a choice, and such a shift would be more likely to occur by having an open debate regarding abortion and the state's responsibility for the provision of welfare services; thereby ensuring that girls can cope with maternity without sacrificing other aspects of their lives-such as education, independence, employment and leisure.

### Main limitations and measures to enhance trustworthiness

Measures were taken in order to strengthen trustworthiness [36]. Regarding credibility - how well the findings had captured the reality being explored - since the first author was living in the area where the research was going on, credibility was enhanced by prolonged engagement in the area and persistent observation. The risk of naivety was minimized by discussion with the other researchers who were not familiar with the setting. The fact that the first author was both an insider (through her daily work in Orellana), and outsider (through her role as researcher), may have influenced the results. On the one side it helped to gain access to participants, on the other side participants' opinions may have been influenced by social desirability bias. Since the first author was also European, the risk of ethnocentrism also has to be taken into account. Credibility was also enhanced by informal debriefing with colleagues and triangulation of researchers. Referent checking was done through informal discussions with providers who were not involved in the study, and by presenting and discussing preliminary results in a workshop with Orellana's service providers and policy makers. In order to stay closer to the text, the original Spanish version was used for coding, and translation into English only took place after repertoires emerged.

Transferability-analytical generalizability - was enhanced by purposely selecting participants based on their ability to contribute to the study question. An effort was made to contextualize the results, so that readers could better evaluate the applicability of the results to other areas. In fact, even if the study is based on a very concrete setting, the results may reflect not only the discourses of Orellana's service providers and policy makers, but also the general discourse of professional stakeholders involved in adolescents' sexual and reproductive health.

Dependability-the ability to respond to issues emerging from the data - was enhanced by following an emergent design, i.e. the interview guides incorporated issues emerging from previous interviews.

Regarding neutrality, we did not claim to be neutral as researchers as in a positivistic paradigm, but in accordance with Lincoln & Guba, we sought neutrality of the data [36]. The researchers' position influenced the research topic and the rights approach, and may have influenced the results. However, in order to avoid biases and stay neutral to data we used the method of "bracketing" [62], which here meant not excluding contradictory findings and not going in-depth into existing theories until we had analyzed the data.

## Conclusions

Policy makers and service providers' interpretative repertoires regarding adolescent pregnancies in Orellana were embedded with gender norms. Adolescence was constructed as an underdeveloped stage, sexuality as negative and dangerous for girls, and adolescent pregnancies as a problem that should be dealt with mainly by health professionals. Those repertoires also idealized motherhood, stigmatized abortion, oriented services towards married women, and idealized marriage and the traditional nuclear family, despite the acknowledgment of the high-levels of violence within such institutions. However, some discordant voices emerged, especially from the youngest policy makers, emphasizing the need for strengthening young people's participation, challenging discriminatory attitudes and actions towards adolescents, and developing policies and programs relevant to the needs of adolescents in Orellana and which address gender-power relations.

An adequate system of reproductive health protection should include the health and educational sector, as stressed by the participants in the study. However, the integration of other relevant sectors, such as those dealing with gender-based violence, might significantly reinforce the integrity and relevance of such system. More respectful and democratic interactions between girls and providers would increase girls' access to services, and empower them treating them as autonomous individuals, and helping them to make their own decisions.

## Competing interests

The authors declare that they have no competing interests.

## Authors' contributions

IG did the data collection (designed interview guides, carried out the individual interviews and moderated the focus group discussions), data analysis (transcribed the interviews, did the coding and further analysis) and wrote the manuscript. MW, MSS and AO participated in the design of the interview guides, closely followed the data analysis (interviews, coding process and identification of interpretative repertoires) and revised the drafts of this paper. All authors have read and approved the final version of the manuscript.

## Pre-publication history

The pre-publication history for this paper can be accessed here:

http://www.biomedcentral.com/1472-698X/10/12/prepub
